# Regression in thin melanoma is associated with nodal recurrence after a negative sentinel node biopsy

**DOI:** 10.1002/cam4.922

**Published:** 2016-09-27

**Authors:** Jill C. Rubinstein, Gang Han, Laura Jackson, Kaleigh Bulloch, Stephan Ariyan, Deepak Narayan, Bonnie G. Rothberg, Dale Han

**Affiliations:** ^1^ Section of Surgical OncologyDepartment of SurgeryYale University School of MedicineNew HavenConnecticut06520; ^2^ Department of Epidemiology & BiostatisticsTexas A&MCollege StationTexas77843; ^3^ Medical OncologyDepartment of Internal MedicineYale University School of MedicineNew HavenConnecticut06520; ^4^ Section of Plastic SurgeryDepartment of SurgeryYale University School of MedicineNew HavenConnecticut06520

**Keywords:** Nodal recurrence, regression, sentinel lymph node biopsy, thin melanoma

## Abstract

Prognostic markers for nodal metastasis in thin melanoma patients are debated. We present a single institution study looking at factors predictive of nodal disease in thin melanoma patients. Retrospective review from 1997 to 2012 identified 252 patients with thin melanoma (≤1 mm) who underwent a sentinel lymph node biopsy (SLNB). Node‐positive patients included positive SLNB patients and negative SLNB patients who developed a nodal recurrence (false‐negative SLNB). Clinicopathologic characteristics were correlated with nodal status and outcome. Median follow‐up was 45.5 months. Twelve of 252 patients (4.8%) were node‐positive including six positive SLNB (2.4%) and six false‐negative SLNB (2.4%) patients. No clinicopathologic factors were significantly correlated with nodal disease. For the six false‐negative SLNB patients, median time to nodal recurrence was 37.5 months. Regression was seen in only 16% of cases, but the rate increased to 60% for false‐negative SLNB cases. Both age (odds ratio [OR]: 1.09, 95% CI: 1.01–1.17; *P* = 0.02) and regression (OR: 8.33, 95% CI: 1.34–52.63; *P* = 0.02) were significantly associated with nodal recurrence after a negative SLNB on univariable analysis. Nodal disease in thin melanoma patients was seen in 4.8% of cases. Although regression was not correlated with nodal metastasis, it was correlated with a false‐negative SLNB. Patients with thin melanoma and regression may need more intensive surveillance after a negative SLNB. Further study is needed to determine if the same immune mechanisms that result in regression in primary tumors also lead to regression in lymph nodes, which may decrease detection of melanoma nodal metastases.

## Introduction

The incidence of new melanoma cases in the United States has been rising at an average rate of 1.4% per year 1. The majority (70%) of new melanoma diagnoses consists of thin lesions (≤1 mm). The prognosis is relatively favorable for patients with thin melanoma, with 10‐year survival rates exceeding 90% 2, 3, 4. However, a subset of thin melanoma patients will experience disease recurrence, not uncommonly greater than 10 years after excision of the primary lesion, and the development of nodal metastasis portends a poorer prognosis 5, 6.

Sentinel lymph node biopsy (SLNB) is recommended to evaluate the draining nodal basins for patients with intermediate thickness melanomas (1 mm to 4 mm) in order to provide powerful staging information 7, 8, 9. However, the use of this staging technique for thin melanomas is debated. Neither the National Comprehensive Cancer Network guidelines nor the Society of Surgical Oncology/American Society of Clinical Oncology guidelines recommend routine use of SLNB in thin melanoma patients, instead advocating for discussion and consideration of SLNB in this population 10, 11.

A number of clinicopathologic markers, including Breslow thickness, Clark level, mitotic rate and ulceration status, have been reported as predictive of SLN metastasis in thin melanoma 12, 13, 14, 15. However, there is no consensus as to which factors if any should be used to determine which patients with thin melanoma are at higher risk for nodal metastasis and should be recommended for SLNB. Other factors have also been evaluated as prognostic markers for SLN disease. For instance, data on regression as a prognostic marker for nodal disease are conflicting, but the vast majority of studies do not identify either the presence or absence of regression as predictive of SLN metastasis 16, 17, 18, 19.

In addition to looking at factors predictive of SLN metastasis, studies have attempted to look at factors that may predict a false‐negative SLNB. The overall reported false‐negative rate (FNR) for SLNB performed for melanoma ranges from 4.0% to 21.0% and is associated with age, gender, primary location, thickness, lymphovascular invasion, and presence of in‐transit recurrence 20, 21, 22, 23, 24, 25, 26. Furthermore, the survival of patients who have a false‐negative SLNB and develop a nodal recurrence generally mirrors the worse survival seen in patients who develop macroscopic nodal disease 20. However, the FNR specifically for SLNB performed in patients with thin melanoma has not been well reported.

Given the continued controversy over selection criteria for SLNB in patients with thin melanoma, and the relatively unknown factors associated with a false‐negative SLNB performed in this specific population, we reviewed our experience with SLNB in thin melanoma patients to evaluate for clinicopathologic characteristics associated with the presence of nodal disease and to identify risk factors for a false‐negative SLNB.

## Methods

After approval was obtained from the Institutional Review Board, a retrospective review was conducted looking at patients referred to Yale University between 1997 and 2012 who were treated for localized melanoma through wide local excision (WLE) and SLNB. For this study, only patients with a thin melanoma, defined as a primary tumor thickness of ≤1 mm, were included. Demographic, clinical, primary tumor pathology, nodal pathology, and outcome data were reviewed. A false‐negative SLNB consisted of patients with a negative SLNB who subsequently developed a nodal recurrence specifically in the dissected nodal basin. Node‐positive patients included both patients with a positive SLNB and patients with a false‐negative SLNB.

Primary tumors were resected with a 1 cm margin. SLNB was performed according to techniques previously described 27, 28. Selection of patients with thin melanoma for SLNB was per the discretion of the surgeon. Patients with a positive SLN were routinely offered completion lymph node dissection (CLND).

All specimens were reviewed by Yale pathology. Primary tumor characteristics were assessed. Ulceration, regression and lymphovascular invasion (LVI) were noted as absent or present. Mitotic rate was recorded per mm^2^ and analyzed as either a continuous variable or grouped as <1 versus ≥1 per mm^2^. Tumor‐infiltrating lymphocytes (TIL) was categorized as brisk, nonbrisk, or absent. LVI was considered positive if lymphatic and/or vascular invasion were reported. Regression was noted as present if there was replacement of tumor with fibrosis and lymphocytic cells, however, data on the extent and depth of regression were not available. Evaluation of lymph nodes obtained through SLNB consisted of serial sectioning, review of hematoxylin and eosin‐stained sections, and review of immunohistochemistry of sections stained with S‐100 and Melan‐A.

Standard descriptive statistics including mean (standard deviation), median (range), frequency, and percentages for discrete variables were provided for baseline variables of interest. For comparing node‐positive with node‐negative patients and for comparing false‐negative SLNB with nonfalse‐negative SLNB patients, the Wilcoxon Rank‐Sum test was used to compare age and Breslow thickness, whereas Pearson's Chi‐square test or Fisher's exact test was used to assess the association for categorical variables. Simple univariable logistic regression modeling was conducted to determine whether a clinical variable was a significant predictor for nodal status or a significant predictor for a false‐negative SLNB. The estimated odds ratio (OR) and 95% confidence interval (95% CI) were provided to measure the effect of the association. The FNR for SLNB was calculated as the number of false‐negative SLNB patients divided by the total number of true positives (positive SLNB patients) plus false‐negative SLNB patients multiplied by 100 [false negatives/(true positives + false negatives) * 100]. The Kaplan–Meier product‐limit method was utilized to analyze overall survival (OS) and melanoma‐specific survival (MSS). The log‐rank test was used to test for differences in OS and MSS between any two groups of interest. A *P*‐value of ≤0.05 was considered statistically significant. All statistical analyses were performed by SAS 9.4 (SAS, Cary, NC).

## Results

### Patients and primary tumor characteristics

**Figure 1:**
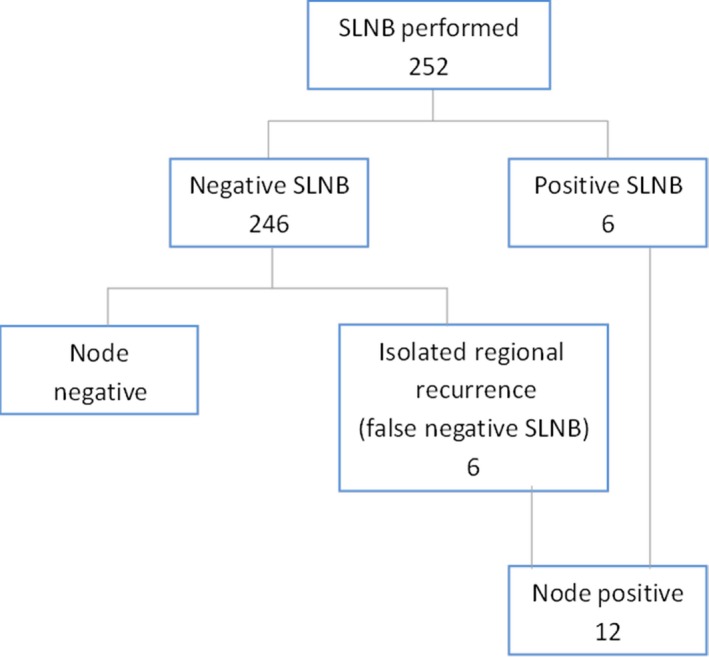
Breakdown of patients. A total of 252 patients with thin melanoma underwent sentinel lymph node biopsy (SLNB). There were six patients with a positive SLNB and an additional six cases of isolated regional recurrence in the dissected basin (false‐negative SLNB), for a total of 12 node‐positive patients.

A total of 252 patients with thin melanoma who underwent WLE and SLNB were included in the study (Fig. 1). Clinicopathologic characteristics of all patients are shown in Table 1. The overall median age was 55.5 years (range 19–86 years) and 52% of all patients were female. Overall median Breslow thickness was 0.9 mm (range 0.4–1.0 mm). Eight percent of cases were ulcerated, whereas regression was seen in 36 of 221 (16%) cases with regression data. A positive SLN was found in six of 252 (2.4%) patients. A CLND was performed in three of the six positive SLN patients with no additional positive nodes found in these three cases.

**Table 1:** Patient demographics and primary tumor characteristics stratified by nodal status (*n* = 252)

Characteristic	All patients (*n* = 252)	Node‐positive (*n* = 12)	Node‐negative (*n* = 240)	*P*
Age (years)				0.27
Median	55.5	60.5	55	
Range	19–86	24–81	19–86	
Gender				0.06
Female	130 (52%)	3 (25%)	127 (53%)	
Male	122 (48%)	9 (75%)	113 (47%)	
Location				0.07
Head & Neck	52 (21%)	5 (42%)	47 (20%)	
Other sites	200 (79%)	7 (58%)	193 (80%)	
Clark level				0.89
II	14 (6%)		14 (6%)	
III	42 (18%)	2 (17%)	40 (18%)	
IV	176 (76%)	10 (83%)	165 (74%)	
V	2 (1%)		2 (1%)	
Ulceration				0.88
No	199 (92%)	10 (91%)	189 (92%)	
Yes	17 (8%)	1 (9%)	16 (8%)	
Growth Phase				0.58
Radial	22 (16%)	1 (10%)	21 (17%)	
Vertical	113 (84%)	9 (90%)	104 (83%)	
Lymphovascular invasion				0.51
No	174 (96%)	10 (100%)	164 (96%)	
Yes	7 (4%)	0 (0%)	7 (4%)	
Tumor‐infiltrating lymphocytes				0.32
Brisk	73 (37%)	2 (20%)	71 (37%)	
Nonbrisk	76 (38%)	6 (60%)	70 (37%)	
None	51 (26%)	2 (20%)	49 (26%)	
Breslow Thickness (mm)				0.91
Median	0.9	0.9	0.9	
Range	0.4–1.0	0.55–1.0	0.4–1.0	
Mitotic rate				0.81
<1/mm^2^	60 (29%)	3 (27%)	57 (29%)	
≥1/mm^2^	145 (71%)	8 (73%)	137 (71%)	
Regression				0.31
No	185 (84%)	8 (73%)	177 (84%)	
Yes	36 (16%)	3 (27%)	33 (16%)	

Node‐positive includes positive sentinel lymph node biopsy (SLNB) patients and patients who developed a nodal recurrence in the dissected nodal basin after a negative SLNB.

The overall median follow‐up was 45.5 months. Of the 246 patients with a negative SLNB, six patients (2.4%) developed a nodal recurrence in the dissected nodal basin during follow‐up. For assessing potential associations between primary tumor characteristics and nodal status, the cohort was divided into node‐negative and node‐positive groups. In total, 12 of 252 (4.8%) patients were node‐positive (six positive SLNB and six false‐negative SLNB patients), whereas 240 patients had no evidence for nodal disease at last follow‐up and were considered node‐negative.

### Predictors of nodal metastasis

As shown in Table 1, node‐positive patients tended to be older with a median age of 60.5 years compared with a median age of 55 years for node‐negative patients (*P* = 0.27). In addition, only 25% of node‐positive patients were female compared with 53% of node‐negative patients (*P* = 0.06). Node‐positive patients tended to have more tumors on the head/neck (42%) compared with node‐negative patients (20%, *P* = 0.07). Overall, regression was seen in 16% of cases, but was seen in 27% of node‐positive cases compared with 16% of node‐negative cases (*P* = 0.31).

Demographic and primary tumor characteristics were correlated with nodal status. No clinicopathologic factors significantly predicted for nodal disease on univariable logistic regression analysis, but female gender (OR: 0.30, 95% CI: 0.08–1.12; *P* = 0.07) and head/neck location (OR: 2.933, 95% CI: 0.89–9.65; *P* = 0.08) trended toward significance as prognostic markers for nodal disease. An additional analysis was performed looking at demographic and pathology factors that were correlated with a positive SLNB, however, no clinicopathologic factors were significant prognostic markers for SLN metastases.

### Predictors of a false‐negative sentinel lymph node biopsy

For the six patients who had a false‐negative SLNB, median time to nodal recurrence was 37.5 months. Four of these false‐negative cases had melanomas ≥0.75 mm, whereas all six patients with a false‐negative SLNB had melanomas that were Clark level IV. Five false‐negative cases had a vertical growth phase, whereas the remaining case had a radial growth phase. Four false‐negative cases demonstrated LVI (data missing for the remaining two patients), whereas brisk TIL was seen in one false‐negative case and nonbrisk TIL was seen in two other false‐negative cases.

As shown in Table 2, patients who had a false‐negative SLNB were significantly older (median age: 69.5 years) compared with patients who did not have a false‐negative SLNB (median age: 55 years, *P* = 0.01). In addition, regression was seen at a significantly (*P* = 0.01) higher rate in patients who had a false‐negative SLNB (3 of 5 patients, 60%) compared with patients who did not have a false‐negative SLNB (33 of 216 patients, 15%). There was no significant difference in median Breslow thickness (0.8 mm vs. 0.9 mm, respectively, *P* = 0.2), ulceration (20% vs. 8%, respectively, *P* = 0.31), mitotic rate, or mean number of sentinel nodes resected (3.5 vs. 3, respectively, *P* = 0.62) between patients who had a false‐negative SLNB and patients who did not have a false‐negative SLNB.

**Table 2:** Test of association between characteristics of false‐negative SLNB and the remainder of the cohort

Characteristic	All Patients (*n* = 252)	False Negative SLNB (*n* = 6)	True‐Positive and True‐Negative SLNB (*n* = 246)	*P*
Age (years)				0.01
Median	55.5	69.5	55	
Range	19–86	58–81	19–86	
Gender				0.08
Female	130 (52%)	1 (17%)	129 (52%)	
Male	122 (48%)	5 (83%)	117 (48%)	
Location				0.44
Head & Neck	52 (21%)	2 (33%)	50 (20%)	
Other sites	200 (79%)	4 (67%)	196 (80%)	
Number of Sentinel Nodes Resected (mean)	3	3.5	3	0.62
Ulceration				0.31
No	199 (92%)	4 (80%)	195 (92%)	
Yes	17 (8%)	1 (20%)	16 (8%)	
Breslow Thickness (mm)				0.20
Median	0.9	0.8	0.9	
Range	0.4–1.0	0.55–1.0	0.4–1.0	
Regression				0.01
No	185 (84%)	2 (40%)	183 (85%)	
Yes	36 (16%)	3 (60%)	33 (15%)	

SLNB, sentinel lymph node biopsy.

On univariable logistic regression analysis, both age (OR: 1.09, 95% CI: 1.01–1.17; *P* = 0.02) and regression (OR: 8.33, 95% CI: 1.34–52.63; *P* = 0.02) were significantly correlated with a nodal recurrence in the dissected nodal basin after a negative SLNB. Of note, none of the six positive SLNB cases demonstrated regression in the primary lesion.

### Recurrence and survival

Of 252 patients, 18 (7.1%) patients experienced disease recurrences, including three isolated local recurrences, six nodal recurrences in patients who had a negative SLNB (one of these patients also developed distant metastasis), and four patients who developed only distant recurrences. The type of recurrence was unknown in five cases. The negative predictive value for SLNB was 97.6%, whereas the FNR was 50% (6 positive nodes missed out of 12 total node‐positive cases).

There were 12 deaths for all 252 patients (4.8%) of which 11 deaths were melanoma‐related. Three of 6 (50%) patients in the false‐negative SLNB group died, and the cause of death was from melanoma in all three cases. The remaining nine deaths were among the 246 patients (3.7%) who did not have a false‐negative SLNB, and all but one of these deaths were melanoma related. All six patients with a positive SLNB were alive with no evidence of disease at last follow‐up after a median follow‐up of 29.4 months.

**Figure 2:**
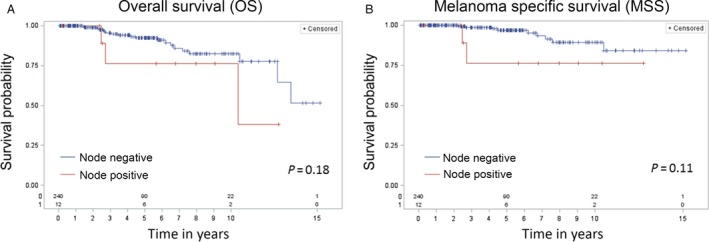
(A) Overall Survival (OS) and (B) Melanoma‐Specific Survival (MSS) comparing node‐positive with node‐negative patients. OS and MSS were worse for node‐positive patients compared with node‐negative patients, however, the differences were not significant (*P* = 0.18 for OS,* P* = 0.11 for MSS).

**Figure 3:**
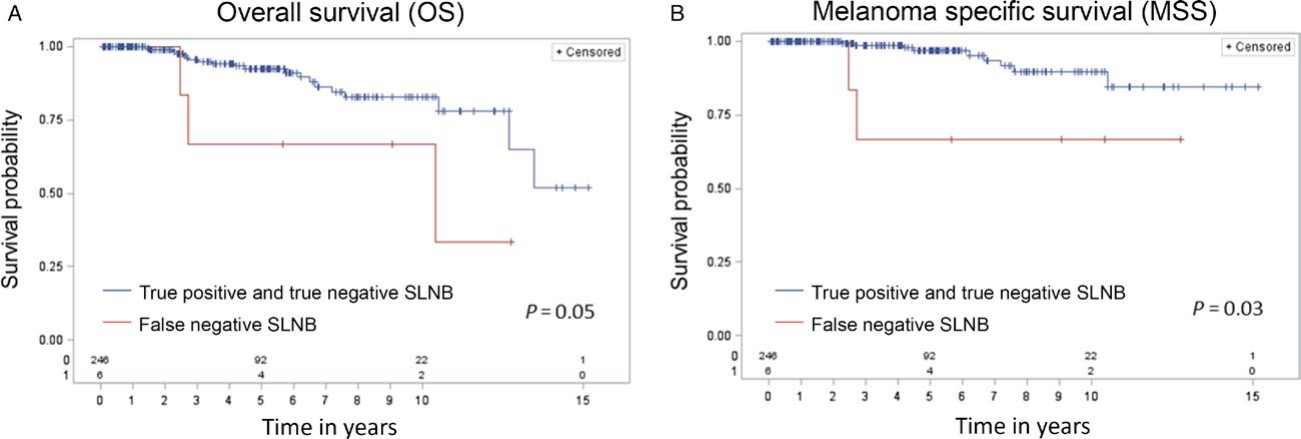
(A) Overall Survival (OS). The six patients with a false‐negative sentinel lymph node biopsy (SLNB) are compared with the remainder of the cohort, demonstrating a significant association between false‐negative SLNB and decreased OS (*P* = 0.05) (B) Melanoma‐Specific Survival (MSS). Comparison of false‐negative SLNB patients with the remainder of the cohort shows a significant association between false‐negative SLNB and decreased MSS (*P* = 0.03).

OS and MSS curves comparing node‐positive and node‐negative patients are shown in Figure 2. Although OS and MSS were worse for node‐positive patients compared with node‐negative patients, the differences were not significant (*P* = 0.18 for OS, *P* = 0.11 for MSS). In Figure 3, OS and MSS curves are shown comparing patients who had a false‐negative SLNB with the remaining 246 patients. OS and MSS were significantly worse for patients who had a false‐negative SLNB compared with patients who did not (*P* = 0.05 for OS, *P* = 0.03 for MSS).

## Discussion

SLNB has become part of the standard of care for intermediate thickness melanomas 7. In fact, for intermediate thickness melanomas, the presence of SLN metastasis was the most powerful predictor of recurrence and death in 10‐year follow‐up data from the MSLT‐I trial 7. Despite increasing numbers of newly diagnosed melanomas ≤1 mm in thickness, indications for SLNB in these patients remain controversial. In a meta‐analysis that included 60 studies, the estimated proportion of thin melanoma patients with SLN metastasis was 4.5% (mean 5%, SD 3.9%) 29. There are a growing number of studies exploring the association of clinicopathologic features such as Breslow thickness, Clark level, mitotic rate, and ulceration with nodal disease in thin melanoma 12, 13, 14, 15. In our study, 4.8% of thin melanoma patients were found to harbor nodal disease. Although our rate of a positive SLN was only 2.4%, the overall nodal metastasis rate was 4.8% which is consistent with the rates of nodal disease in thin melanoma reported in other studies. None of the evaluated clinicopathologic factors were significantly associated with the presence of nodal metastasis, but there was a trend toward increased risk with male gender and tumor location on the head and neck. Given the small number of patients with a positive SLN or nodal disease overall, it is not surprising that we were unable to demonstrate significant correlations between clinicopathologic factors and nodal metastasis. Despite this, it should be noted that all patients with a positive SLN had melanomas ≥0.75 mm in thickness, and of the six patients with a false‐negative SLNB, four cases had melanomas ≥0.75 mm in thickness. Combining the patients with a positive SLNB and a false‐negative SLNB yields a total node positivity rate of 5%. It may be reasonable to use a risk threshold for nodal disease of 5% to offer nodal staging, particularly in patients with melanomas ≥0.75 mm in thickness, but it is not possible to make definitive conclusions about indications for SLNB in thin melanoma based on these data.

The prognostic significance of a positive SLN in thin melanoma patients is unknown. Previous studies have shown that median time to recurrence for thin melanoma was over 38 months 30. In this study, all patients with a positive SLN were alive, but with a median of only 29.4 months of follow‐up, an impact on survival may not yet be seen. The study by Wright et al. showed that for thin melanoma patients, it takes approximately 4–5 years before the MSS effect of SLN status is seen 31. Furthermore, in looking at all node‐positive patients, there was a trend showing worse OS and MSS in node‐positive patients when compared with node‐negative patients. It is likely that with a larger number of patients and with longer follow‐up, a significant difference in OS and MSS could be seen between node‐positive and node‐negative patients.

In accord with the literature, the presence or absence of regression in this cohort was not correlated with node positivity. Of the six patients with a positive SLNB, none demonstrated regression in the primary lesion. However, there was an association between a false‐negative SLNB and the presence of regression in the primary lesions. Specifically, 60% of false‐negative SLNB cases that had regression data demonstrated regression (5 of 6 false‐negative cases had regression data with 3 of these 5 cases having regression), and this was correlated with a nodal recurrence after a negative SLNB on univariable logistic regression analysis.

Regression in melanoma is an immunologic process characterized by lymphocytic infiltration causing the spontaneous disappearance of tumor cells, leading to separation of the tumor into distinct islands with intervening areas of nontumor containing fibrotic tissue. The significance of regression for overall prognosis has long been disputed and remains unclear 32, 33, but here we describe an association between the presence of regression and an increased risk for a false‐negative SLNB. Interestingly, in a 2008 study, Morris et al. identified an increased overall risk of recurrence in those patients *without* regression; however, there was neither an increased nor decreased risk of recurrence in the nodal basin (i.e., false‐negative SLNB) on the basis of regression in the primary 16. In contrast, none of the true‐positive SLNB cases in our study demonstrated regression. We postulate that the presence of regression may make detection of lymph node metastasis more difficult. It is possible that the same immune mechanisms that cause regression in the primary tumor may also cause regression of metastatic deposits in lymph nodes, thereby decreasing detection. It is also possible that the presence of regression led to under‐appreciation of the true Breslow depth in the primary. Regardless of these potential confounders, the data suggest that patients with thin melanoma, regression, and a negative SLNB may require more intensive surveillance since they appear at higher risk for a nodal recurrence.

Older age was also noted to correlate with an increased risk of a false‐negative SLNB. This is in accord with previously published results and has been postulated to relate to decreasing lymphatic function with increasing age 34, 35. Indeed, lymphatic dysfunction has been demonstrated by measurement of radiocolloid transit to and uptake within sentinel lymph nodes, with the authors concluding that age‐related alterations in lymphatic function may lead to alternate metastatic patterns with decreased nodal positivity 35. These findings suggest that older patient age may necessitate special considerations in interpretation of SLNB results.

Valsecchi et al. reported in a meta‐analysis that the FNR reported for SLNB performed for melanoma ranged from 0 to 34%, and the weighted summary estimate was 12.5% 26. A recent study by Lee et al. analyzed 2,986 melanoma patients (including 1151 under 1 mm in Breslow depth) and found that risk factors for a false‐negative SLNB include male gender, tumor location on the head and neck, and the presence of local or in‐transit recurrence 36. Another large study, including 515 patients, corroborated the increased risk of a false‐negative SLNB based upon location in the head and neck and male gender, and showed that increasing Breslow thickness and the presence of ulceration were additional risk factors for a false‐negative SLNB; regression was not found to be associated with a false‐negative result 34. The FNR in this study is relatively high, but the population of patients may be unique due to the relatively high prevalence of regression in the false‐negative cases. If cases with regression are excluded, there would be a total of nine cases with nodal disease with three cases having a false‐negative SLNB, resulting in a FNR of 33.3%. This value would be more consistent with the range reported for FNR by Valsecchi et al. 26.

The major limitations of this study are the small sample size and relatively short follow‐up. In addition, further study is needed to determine if the association between regression and a false‐negative SLNB observed in this study holds in a larger cohort of thin melanoma patients and on multivariable analysis, particularly since nodal recurrence may also be associated with other factors such as Breslow thickness, ulceration status, and presence of LVI. It may be necessary to distinguish between different mechanisms and phases of regression in order to draw clinically meaningful conclusions 37. Furthermore, while there are many studies investigating the impact of regression in the primary tumor, we are unaware of any such studies evaluating for the presence of regression in the nodal basin. If additional investigation reveals that the same immune mechanisms driving regression in the primary tumor are also at play in the lymph nodes, it is possible that regression of metastatic deposits in lymph nodes may make it more difficult to detect these regressed metastatic deposits by SLNB. Thus, it may be that thin melanoma patients who display regression merit more intensive surveillance after a negative SLNB.

## Conclusions

Approximately 5% of patients with thin melanoma will harbor nodal disease. Regression is associated with nodal recurrence after a negative SLNB in this population. Therefore, patients with thin melanoma and regression may need more intensive surveillance after a negative SLNB. Further studies are needed to evaluate the indications for SLNB in thin melanoma patients and to investigate whether the same immune mechanisms that lead to regression in the primary also do so in the lymph nodes, potentially affecting the ability to detect lymph node metastases.

## Conflict of Interest

The authors have no funding sources or conflicts of interest to disclose.
